# The President’s Address

**Published:** 1893-06

**Authors:** James T. Martin


### THE PRESIDENT'S ADDRESS.

### A PHILOSOPHICAL EXPOSÉ OF THE LAW OF SIMILARS.

Read before the California State Homœopathic Society, May 10th, 1893, by James T. Martin, M. D.

It has always seemed to me that there is a philosophical explanation of the action of the law of similars and their natural corollary, potentiation, and the more I study the subject the more thoroughly I am convinced that the explanation is not only practical but competent to cover the whole ground.

No one who is at all schooled in natural philosophy will for a moment admit that anything ever happens by chance; and whatsoever change occurs, whether development to full fruition or decay to complete disintegration, does so in accordance with the definite and fixed laws of its existence. Our living, breathing, nourishing, and all the functions of organic life as well as those of decay are mandates of law, and the inorganic as well must obey its behests. Law is force: regular, constant, sustaining, and its phenomena are manifest everywhere throughout nature awaiting our careful observation.

At present we will consider only the law governing the changes in organic bodies. Biologically, organic bodies are for the most part made up of the following elementary substances—carbon, hydrogen, nitrogen, and oxygen. Of these carbon is one of the most stable of all known substances. It is isomeric in its properties, and is therefore capable of existing in several different states with different arrangements of its atomic elements.

The other three, while the most unstable of all known elements, and possessing greater molecular activity than any other known substances, are also isomeric to a certain degree. It is a peculiar property of these elements that they communicate their molecular activity more or less to all the compounds into which they enter. It is not here necessary to enter into a discussion of the various intricate chemical combinations into which these elements arrange themselves, nor to discuss the various chemical formulæ entering into their compounds. But suffice it to say that their principal compounds with which we have to deal are crystalloids and colloids.

The colloids are of two kinds, nitrogenous and non-nitrogenous. They are very minute jelly-like bodies, inert, very unstable, and possessing a high degree of molecular mobility. They do not take on chemical change easily or give up their atoms to form other compounds readily. And yet they are stable enough to maintain their integrity under ordinary conditions to which the body may be exposed. This includes quite a wide range of temperature, different atmospheric states, and so on.

The nitrogenous colloidal compounds are imbedded in the non-nitrogenous compounds in the cells of organic bodies. They are the centres of force or energy, and are dynamical in their function and the centre and source of the dynamical forces of the body.

They are also permeable by liquids, not capable of osmose, and do not lose their integrity when so permeated. The crystalloids are statical and capable of osmose; but it is thought that the colloids take nourishment from the crystalloids by dialysis and appropriate energy and force, while the *débris* or used-up materials are set free.

The colloid bodies do not act so well when isolated or alone as they do when near or in conjunction with other colloids. Their atomic motion, which seems to be reciprocally communicated from one to the other, is in unison and is uniform throughout the body. This is the vital-force that has been talked about so much. It is dynamic both in its origin and expression.

The extreme mobility of which these bodies are possessed renders them very sensitive to the action of any incident force whatever, such as light, heat, cold, drugs, disease, and so on; any one of these acting for any length of time will produce disturbances in the colloidal bodies and *de facto* disturbances in the dynamical forces of the body which either exceeds or comes short of that which is natural, and we have various phenomena expressed known as disease—that is, the natural normal equilibrium of the body has been so disturbed that we have an unnatural or diseased state, and the question that now confronts us is how we are to put this unnatural state to rights, and what is the means necessarily employed to reduce the disturbed equilibrium to its normal motion, which we recognize as health. These are all forces in nature and are just as amenable to the laws of physics as any other forces from whatsoever source.

It is the law of mechanics * that "If a body, which some force has set in motion, impinges upon another body, it imparts motion to it, and is therefore itself a force." This is as true of absolute force as it is of bodies representing force applied, and

* Olmstead.

may be applied to the forces maintaining organism as follows: If the vital-force is impinged upon by any incident force it partakes more or less of the nature of that incident force, and itself expresses more or less of the character of that force, and hence we have a deviation from the normal standard, health. Now, it makes no difference from what standpoint you consider life, whether theological, psycological, physiological, or biological, it must have as a basis of existence and expression the atomic motion of the colloidal bodies, which is the "vital-force." This will have to be recognized as a distinct force in nature, subject to the same modifications, restrictions, and influences as other natural phenomena and governed in the same way. This being the case, we have only to bring the principles of natural philosophy to our aid to understand its meaning aright; and when our knowledge is clear on this point we are able to bring to our aid other natural forces that will control or modify the perverted vital-force as may be required.

On page 94, *Principles of Biology*, Mr. Herbert Spencer arrives at this conclusion: "That organic matter is specially sensitive to surrounding agencies; that in consequence of the extreme instability of the compounds it contains, minute disturbances can cause in it large amounts of redistributions, and that during the fall of its unstably arranged atoms into stable arrangements there are given out proportionately large amounts of motion. We saw that organic matter is so constituted that small incident actions are capable of initiating great reactions; setting up structural modifications, and liberating large quantities of power." Let the indicated remedy be the force incident upon the organism, and you will initiate reactions, set up structural changes, and liberate power all in proportion to the directness and quality of the force incident.

Again on page 289, after speaking of the influence of light on glass and other inorganic substances, Mr. Spencer says: "We are obliged to conclude that the excessively unstable units of which organisms are built, must be sensitive in a transcendent degree to all the forces pervading the organisms composed of them—must be tending ever to readjust not only their relative positions, but their molecular structures into equilibrium with that force."

I might add, were it not for this fact it would be extremely difficult to produce any impression whatever on the organism with drugs or any other force. It is hardly possible that there is any one with sufficient temerity to assert that disease is not a natural force, and that it is not governed by the same general laws as other natural forces. This, then, being the case, we have distinctly three forces to deal with—that is, the vital-force, disease-force, and drug-force.

Disease manifests itself in various forms; each particular form is constant in the phenomena it presents, so much so that we have learned to recognize them by a name distinctive of the kind of manifestation presented. These manifestations, of whatever name, have been produced by some incident force acting upon the vital-force until it expressed the nature of the incident force, and you have some particular disease manifested, while the natural equilibrium of the body is obscured.

This is general and not used in any restricted sense whatever, and can be applied to any morbific force. It is not necessary for me to make this in *more* general terms than that the weaker force must yield to the stronger and itself express the individuality of the latter instead of its own, and in accordance with the principle before stated, any force may bring about organic changes and reduce the collodial bodies with stable units to unstable units and thereby destroy their natural functions.

This is as much in accordance with the law of Homœopathy as it is with natural philosophy. There never has been a time, and there never will come a time, when the truth will be proved false by any course of reasoning or scientific experiment whatever. If there is any proving, it will be either a demonstration *of* the truth or the falsity of the so-called scientifie experiment.

In the development of our materia medica, we have included a wide and varied range of drugs. Very many of them, in fact, all that have been well proved, have been given the practical test of experience and found to operate directly in the line expected of them.

On page 14, *Principles of Biology*, after discussing the practical application of mechanical principles to atomic motion, Mr. Spencer says: "Other things being equal therefore the molecular mobility of atoms must decrease as their mass increases, and so there must result that general progression, we have traced, from the higher molecular mobility of those large atomed substances into which they are ultimately compounded. Applying to atoms the mechanical law which holds of masses, that since inertia and gravity increase as the cubes of their dimensions, cohesion increases as their squares.

"The self-sustaining power of a body becomes relatively smaller as its bulk becomes greater. It might be argued that these large aggregate atoms which constitute organic substances, are mechanically weak, are less able than simpler atoms to bear without alteration, the forces falling upon them. That very massiveness which renders them less mobile enables physical forces acting on them more readily to change the relative positions of their component atoms." The truth here in this quotation first stated may be re-arranged as follows without doing violence to its integrity, and we have, therefore, the molecular mobility of atoms, which must increase as their mass decreases. This is a general statement, and must hold true howsoever far the decrement of the mass is pursued. Applying this deduction to the homœopathic attenuation it necessarily follows as a logical conclusion that as the mass of the drug decreases its molecular mobility increases, and also that it has greater resisting power than when in the mass; hence we might infer that the crude drug when taken into the body has not as much power for mischief as the same amount of the same drug properly attenuated. The simpler the atoms the greater their power. A person might take five grains of Calomel, which would only produce catharsis, while one-tenth of a grain of the same triturated and taken would likely produce salivation and other physiological effects of the drug, and as we have before stated as the mass of the drug becomes less its atoms become more active. This fact is also borne out by the experience of all who have made proper practical tests of drugs so treated.

Dr. G. Jeager in his neural analysis has found this to be true up to the 1,000th potency, which was the extent of his experiments. He first measured the normal nerve-force with his chronoscope and then at stated intervals administered different drugs in different attenuations and measured the increments of molecular motion after each dose until he reached the limit of his experiment. In every instance he noted the difference between the normal markings of his instrument and those made after taking each different attenuation of medicine, and found that the increments of nerve-force grew greater as the attenuation became higher. So as accurate measurements are made of drug-force, the theory of attenuation more clearly corresponds to the inductions of biology.

And as is the case with all other elements in nature each (drug) substance has an atomic motion peculiar to itself which is the expression of the force contained in the drug itself, and while there are many characteristics of its power in any one drug that may be common to many drugs, yet there are always more or less phenomena which are peculiar to that individual alone that are produced only by that drug acting upon the vital-force in the healthy human body. This peculiarity is sufficiently marked that we may recognize each individual by the special manifestations present, and so far as our understanding goes this may be predicated of all drugs, of which we have definite knowledge. Of the three forces of nature with which we have to deal the vital-force has become somewhat modified by the action of the disease-force which was incident upon it, and hence you have the expression of the particular kind of disease that the organism is suffering from. Now to combat the latter we have the drug-force whose peculiarities you have already become acquainted with after a long series of careful experiments. These forces are subject to and governed by the same physical laws as other natural forces; hence, from the data given above and upon general philosophical principles the action of the drug over the disease is easy of explanation. Whenever an atomic force or motion comes in contact with or impinges upon another atomic force or motion with similar increments similarly placed, they will vibrate in unison as one and the same force with a possible and perceptible additional increase of power. Unless this is pursued to a considerable extent the increment of force is hardly noticeable, but when the incident force is much greater than the other it will obscure the original force to such an extent that nothing but the incident force would be easily perceptible.

In whatever organism disease may occur there are always certain phenomena expressed peculiar to that individual alone, and we have learned after a long series of practical tests under the most varied circumstances that the remedy whose action is nearest in accord with these particular manifestations will uniformly cure quicker, more surely than any other, and with the least possible harm to the individual; and we are by no means overstepping the bounds of physical law when we confidently assert that the vital-force which has been impinged upon by disease and thereby changed from its normal expression can be more easily set right by the drug that can, and does, vibrate in unison with it by virtue of its similarity of motion than by any and all others combined.

This unison of motion adds strength to the failing vital-force and gives it power to overcome the invading force, and therefore restore the normal equilibrium. In other words, the drug-force must be of such a nature that it can get into equilibrium with the vital-force or it can have no power in the restoration of normal equilibrium. This cannot and will not take place unless the similar remedy is selected, for none other can vibrate in unison with the vital-force and give to it the necessary power to overcome disease.

